# Efficacy of Aloe Vera Supplementation on Prediabetes and Early Non-Treated Diabetic Patients: A Systematic Review and Meta-Analysis of Randomized Controlled Trials

**DOI:** 10.3390/nu8070388

**Published:** 2016-06-23

**Authors:** Yiyi Zhang, Wen Liu, Dan Liu, Tieyun Zhao, Haoming Tian

**Affiliations:** 1 Department of Endocrinology and Metabolism, West China Hospital, Sichuan University, Chengdu 610041, China; tibby5211@gmail.com (Y.Z.); danliu565@sina.com (D.L.); zty112356@gmail.com (T.Z.); 2 Phase I Clinical Research Unit, West China Hospital, Sichuan University, Chengdu 610041, China; wenliu112@126.com

**Keywords:** aloe vera, prediabetes, randomized controlled trials, meta-analysis

## Abstract

The aim of this study was to evaluate evidence for the efficacy of aloe vera on managing prediabetes and early non-treated diabetes mellitus. We performed a systematic search of PubMed, Embase, and Cochrane Central Register of Controlled Trials until 28 January 2016. A total of five randomized controlled trials (RCTs) involving 415 participants were included. Compared with the controls, aloe vera supplementation significantly reduced the concentrations of fasting blood glucose (FBG) (*p* = 0.02; weighed mean difference [WMD]: −30.05 mg/dL; 95% confidence interval [CI]: −54.87 to −5.23 mg/dL), glycosylated hemoglobin A1c (HbA1c) (*p* < 0.00001; WMD: −0.41%; 95% CI: −0.55% to −0.27%), triglyceride (*p* = 0.0001), total cholesterol (TC) (*p* < 0.00001), and low density lipoprotein-cholesterol (LDL-C) (*p* < 0.00001). Aloe vera was superior to placebo in increasing serum high density lipoprotein-cholesterol (HDL-C) levels (*p* = 0.04). Only one adverse event was reported. The evidence from RCTs showed that aloe vera might effectively reduce the levels of FBG, HbA1c, triglyceride, TC and LDL-C, and increase the levels of HDL-C on prediabetes and early non-treated diabetic patients. Limited evidence exists about the safety of aloe vera. Given the small number and poor quality of RCTs included in the meta-analysis, these results are inconclusive. A large-scale, well-designed RCT is needed to further address this issue.

## 1. Introduction

Diabetes mellitus is a group of metabolic disorders that is one of the major global health threats to humans due to its increasing prevalence, disabling complications, and chronic course [1]. Both impaired glucose tolerance and impaired fasting glucose are called “prediabetes”, which does not reach the diagnostic cutoff values that would precipitate a diagnosis of type 2 diabetes mellitus (T2DM) [2]. It is predicated that approximately 5%–10% of the prediabetic population would suffer from diabetes after approximately a year, and the number of prediabetes patients could reach up to 470 million globally by 2030 [3]. Thus, preventing or delaying the onset of clinical T2DM in prediabetic subjects is a reasonable way to combat the diabetes epidemic and to lessen healthcare costs. Current medications to control blood glucose may have dangerous side effects over time, such as increased risk of liver toxicity, weight gain, and cardiovascular diseases [4]. Therefore, it is not surprising that complementary medicines and natural products, such as oats, are gaining increasing popularity among patients with hyperglycemia, due to affordability and fewer side effects [5]. Many traditional remedies for diabetes mellitus use plant sources, and over 400 species were reported to display hypoglycemic effects, although few of them have been evaluated [6,7].

Aloe is a succulent plant belonging to the Liliaceal family, of which there are more than 200 species found worldwide [8]. Aloe vera is a common name for *Aloe barbadensis*, which is the most widely-used species of aloe. Aloe vera has had applications in health and cosmetic products for many centuries, as well as anti-tumor, antioxidant, anti-inflammatory, and laxative properties [9,10]. It includes over 75 active ingredients that contain enzymes, vitamins, sugars, minerals, lignin, amino acids, and salicylic acid, and most of the constituents appear to be of biological importance in curing diseases [11].

The hypoglycemic effects of aloe vera have been investigated by various researchers. However, there is no consensus on the beneficial effects of aloe vera supplementation in the preventing or improvment of metabolic-syndrome-related disorders [12]. Some studies with rodents have demonstrated the hypoglycemic effect of aloe vera, whereas other studies have shown no significant effects [13,14]. Similarly, there is growing evidence that aloe vera derived extracts showed a preventive effect against insulin resistance and a lipid-lowering effect; however, there is still a great deal of controversy about these data, which have made it difficult to draw any definitive conclusions [14,15]. Therefore, we conducted a meta-analysis to quantitatively summarize and critically evaluate the evidence from randomized clinical trials (RCTs) involving the use of aloe vera as a hypoglycemic supplement.

## 2. Materials and Methods 

### 2.1. Search Strategy

We searched the following three electronic databases from their inception until 28 January 2016 for the identification of studies: PubMed, Embase and Cochrane Central Register of Controlled Trials. Keywords for databases searching were: (“prediabetes” OR “pre-diabetes” OR “prediabetic state” OR “hyperglycemia” OR “borderline diabetes” OR “impaired glucose tolerance” OR “impaired fasting glucose” OR “diabetes prevention” OR “prevention of diabetes” OR “diabetes mellitus” OR “diabetes mellitus type 2” OR “non-insulin dependent diabetes mellitus”) AND (“Aloe” OR “Aloe vera” OR “Aloe barbadensis” OR “Aloe barbadensis extract” OR “Aloe vera extract”). All of the indexed studies were retrieved, and the reference lists of the identified publications were reviewed for additional pertinent studies. No language restriction was applied for searching. The literature search and study selection were carried out independently by two reviewers (Y.Z. and W.L.) with a standardized approach. Any inconsistencies were resolved by consultation with a third reviewer (D.L.).

### 2.2. Inclusion Criteria

The diagnostic of prediabetes or T2DM was based on the definitions described by the World Health Organization [16] or the American Diabetes Association [4]. Patients were not limited by age, sex, race, or body size. The inclusion criteria were as follows: (1) randomized controlled trials; (2) studies were included irrespective of whether or not they incorporated lifestyle changes into their trial regimen; (3) studies involving the use of aloe vera as part of a combination product or treatment package were excluded from the systematic review; (4) no history of any hypoglycemic medication use; (5) no history of other diseases like coronary heart disease, stroke, cancer, hepatic disorder, a chronic inflammatory condition, or psychiatric disorders; and (6) primary outcomes reported included at least glucose and/or lipid profile.

### 2.3. Data Extraction and Quality Assessment

One reviewer screened the titles and abstracts of the RCTs that we identified. Full text articles were obtained for those trials that fulfilled the inclusion criteria or for which sufficient information was given. Two reviewers (Y.Z. and W.L.) independently extracted data from trials that met the inclusion criteria on an Excel spreadsheet; any discrepancies in extracted data were resolved by a group discussion and consensus and final arbitration by the Cochrane editorial base. Attempts were made to seek further information from the authors of the original studies if data were unclear or incomplete. The methodologic quality criteria of randomization, allocation concealed, blinding, and intention-to-treat (ITT) analyses were graded as adequate, inadequate, and unclear. Studies were categorized as double blinding, single blinding, or unclear.

### 2.4. Statistical Analysis

In our meta-analysis, glucose metabolism, such as fasting blood glucose (FBG), insulin, and glycosylated hemoglobin A1c (HbA1c), were considered primary outcomes. Because diabetes was strongly associated with an increased risk of cardiovascular disease, and often coexists with dyslipidemia, other secondary outcomes included lipid profile, such as triglyceride, total cholesterol (TC), low density lipoprotein-cholesterol (LDL-C), and high density lipoprotein-cholesterol (HDL-C). All outcomes extracted from the literature were continuous data.

Review Manager soft-ware 5.2 provided by the Cochrane Collaboration was used for the meta-analysis. For continuous data, weighted mean differences (WMDs) or standardized mean differences (SMDs) with their 95% confidence intervals (95% CIs) were calculated. Analyses were separately performed for each outcome. Heterogeneity across studies was assessed by the Cochrane’s Q-test. The fixed-effects model was used to calculate the total effect size where Cochrane’s Q-test *p* > 0.10 and the *I*^2^ statistic *I*^2^ < 50% indicated statistical homogeneity. If heterogeneity of *p* < 0.10 or *I*^2^ > 50% was found among the trials, a random-effects model was chosen. Two-tailed *p*-values ≤ 0.05 or 95% CIs not containing 0 (WMD) were considered statistically significant. Publication bias was not assessed because the number of the included trials was less than 10.

## 3. Results

### 3.1. Eligible Studies and Baseline Characteristics

**Figure 1:**
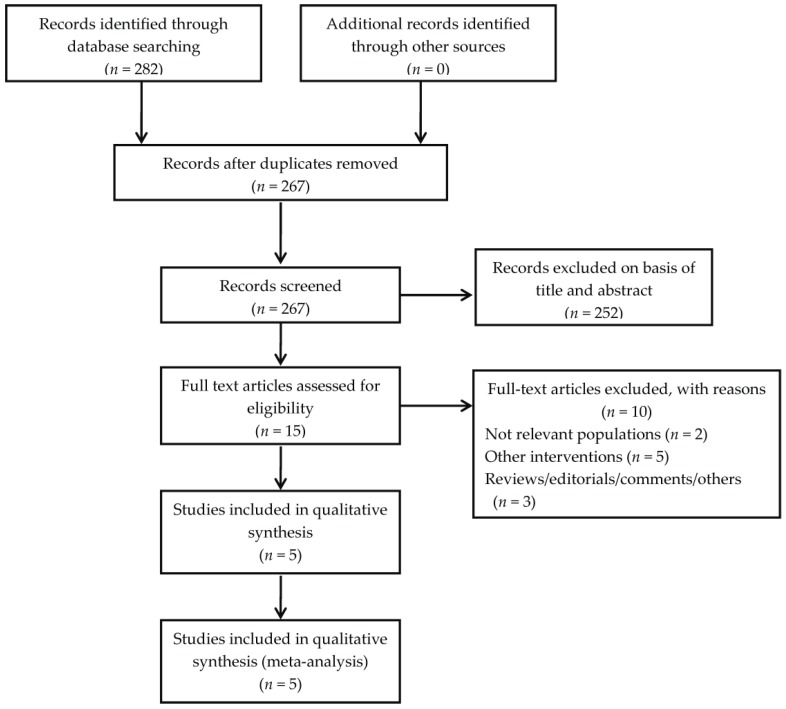
Flow diagram for study identification.

We identified 282 records on aloe vera after searching the three databases mentioned above. Finally, a total of five randomized controlled trials [17,18,19,20,21] from 1996 to 2016 containing 415 participants were included (Figure 1). The key characteristics of these studies are outlined in Table 1.

Most of the included RCTs had flaws in the reporting of their methodology. Although randomization was declared in all studies, only two trials reported how the randomization was conducted [19,21], and none of the studies reported adequate allocation concealment. Three of five studies turned out to be double-blinded [18,19,21], and information relating to withdrawal/dropout was reported adequately in three trials [17,19,21]. ITT analysis was conducted in only one study [19].

All included RCTs involved overweight and/or obese participants, except for one study that did not report [17]. Only one adverse event was reported [19]. Participants in two RCTs [18,21] were allowed to continue their normal lifestyle, whereas the remaining studies did not report this information. The duration of follow-up ranged from 6 to 12 weeks. According to comparators used in these studies, our meta-analysis was divided into two parts.

### 3.2. Meta-Analyses of Primary Outcomes

**Figure 2:**
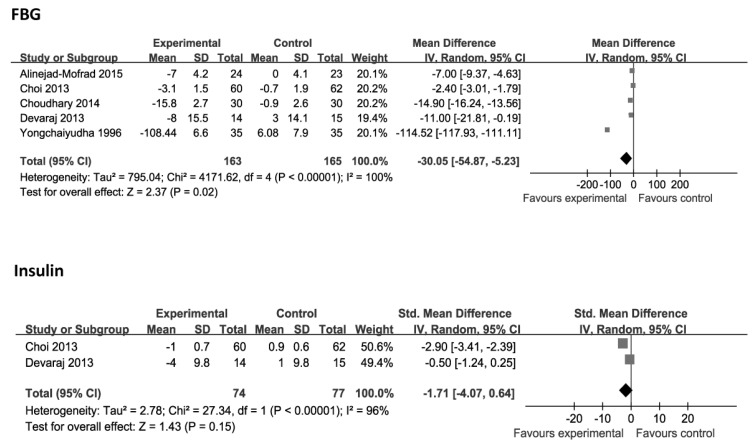

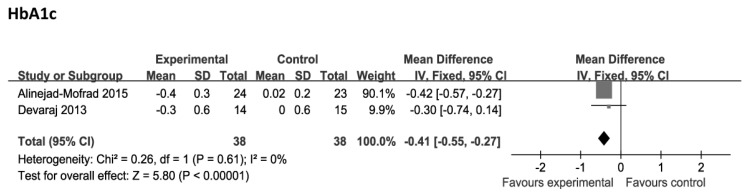
Forest plot of studies that evaluated the effect of aloe vera on primary outcomes compared with placebo. Each block represents a study. Size of square is proportional to the precision of the estimate. Each square represents the weighted mean difference (WMD) or standardized mean difference (SMD) for each study with 95% confidence interval (CI) indicated by horizontal line. FBG, fasting blood glucose; HbA1c, hemoglobin A1c.

The changes in FBG were evaluated in the five studies [17,18,19,20,21], which included 328 cases. However, significant heterogeneity was observed among these studies (*p* < 0.00001, *I*^2^ = 100%). The random effects model of meta-analysis was used to combine the effect size. Aloe vera was superior to placebo in reducing FBG levels (*p* = 0.02; WMD: −30.05 mg/dL; 95% CI: −54.87 to −5.23 mg/dL). Two studies compared the effects of aloe vera and placebo on insulin [18,19]. There were no significant changes in the concentration of insulin (*p* = 0.15; SMD: −1.71; 95% CI: −4.07 to 0.64), with heterogeneity existing among these studies (*p* < 0.00001, *I*^2^ = 96%). The changes in HbA1c were evaluated in the two studies [18,21]. Analysis of aggregated data showed a significant reduction in HbA1c (*p* < 0.00001; WMD: −0.41%; 95% CI: −0.55 to −0.27%), without evidence of heterogeneity (*p* = 0.61, *I*^2^ = 0%; Figure 2).

### 3.3. Meta-Analyses of Secondary Outcomes

**Figure 3:**
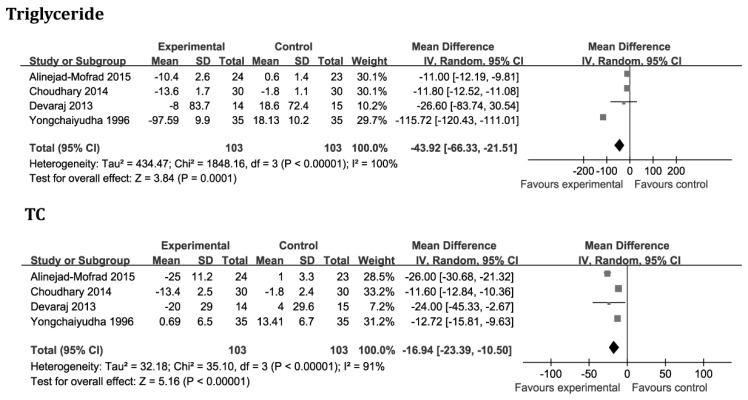

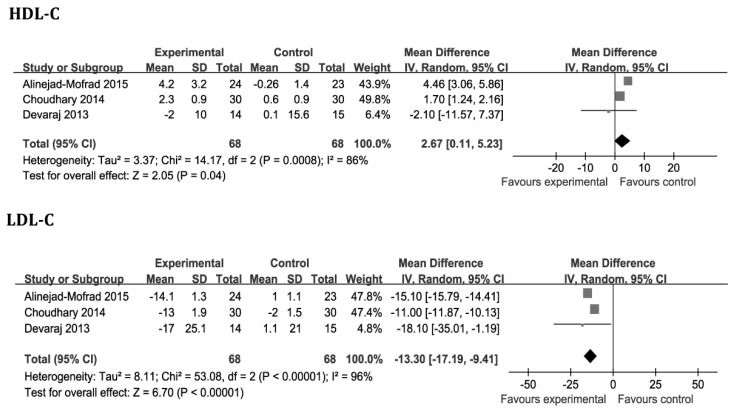
Forest plot of studies that evaluated the effect of aloe vera on secondary outcomes compared with placebo. See Figure 2 for the legend of symbols used. TC, total cholesterol; HDL-C, high density lipoprotein-cholesterol; LDL-C, low density lipoprotein-cholesterol.

Four studies [17,18,20,21], which included 206 cases, compared the effects of aloe vera and placebo on triglyceride and TC levels. After analysis of the aggregated results, we found that aloe vera was superior to placebo in reducing serum triglyceride and TC levels (*p* = 0.0001; WMD: −43.92 mg/dL; 95% CI: −66.33 to −21.51 mg/dL and *p* < 0.00001; WMD: −16.94 mg/dL; 95% CI: −23.39 to −10.50 mg/dL, respectively), although heterogeneity existed among these studies (*p* < 0.00001, *I*^2^ = 100% and *p* < 0.00001, *I*^2^ = 91%, respectively). The changes in HDL-C and LDL-C levels were evaluated in the three studies [18,20,21]. Aloe vera was superior to placebo in increasing serum HDL-C levels (*p* = 0.04; WMD: 2.67 mg/dL; 95% CI: 0.11 to 5.23 mg/dL), and reducing serum LDL-C levels (*p* < 0.00001; WMD: −13.30 mg/dL; 95% CI: −17.19 to −9.41 mg/dL). High heterogeneity was detected with HDL-C and LDL-C variables (*p* = 0.0008, *I*^2^ = 86% and *p* < 0.00001, *I^2^* = 96%, respectively; Figure 3).

## 4. Discussion

This study is the first meta-analysis regarding the effects of aloe vera for prediabetes and non-treated diabetic patients that has been conducted to synthesize the results from independent randomized controlled studies to draw an overall conclusion. Although significant differences between groups were found for most parameters, the limited evidence reveal a statistically significant difference in reducing serum FBG and HbA1c levels favoring aloe vera over placebo. In addition, aloe vera has also been shown to reduce the levels of triglyceride, TC and LDL-C, and increase the levels of HDL-C.

Our findings are consistent with some animal studies [13,14]. Several mechanisms may be involved in the association between glucose-lipid metabolism and aloe vera. It could be due to the efficacy of high molecular weight polysaccharides or phytosterols isolated from aloe vera gel in enhancing glucose transport by modulating the proximal and distal markers involved in glucose uptake and reducing serum concentrations of cholesterol by reducing the absorptions of cholesterol from the gut. Another theory is that the aloe vera extract can lower the level of blood glucose and lipid in diabetic rats by reducing toxic effects of fat in the liver to improve sensitivity of cells to insulin [14,22,23]. It has also been hypothesized that normalization of plasma lipid status by aloe vera may be explained by its ability to suppress adipogenic gene expression, increased clearance and decreased production of the major transporters of endogenously synthesized cholesterol and triglycerides [14]. Additionally, some researchers believe that aloe vera reduce body fat and improve insulin sensitivity by activating adenosine monophosphate-activated muscle protein kinase, which is important in the regulation of glucose and lipid metabolism [24].

Although some reviews have recently been published about the hypoglycemic effects of aloe vera, the quality of these reviews was limited, and they did not present specific methods on data extraction or assessment of heterogeneity. The following factors have strengthened this meta-analysis. First, studies were included or excluded according to strict criteria. Next, the meta-analysis offered an up-to-date and complete overview of all RCTs involving the efficacy of aloe vera supplementation in managing prediabetes and early non-treated diabetic patients, because it was the result of an extensive search, including gray literature and unpublished studies. 

There are several limitations should be considered before recommending the findings of this review to clinicians. First, our analysis was based on small number of trials and the backgrounds of patients varied, which would result in low statistical power and the publication bias could not be excluded. Second, insufficient data were available. Insulin or glycosylated HbA1c was not available in the majority of studies, thereby limiting the reliable results. Next, some studies [17,18,20] did not examine or report whether or not blinding requirements were fully met and allocation concealments were fully achieved. ITT analysis was performed in only one study [19]. Hence, selective bias and measurement bias may have existed in the trails. Finally, significant between-study heterogeneity was detected for most of the variables assessed. Three different aloe vera-based preparations were manufactured in five different regions, Thailand, United States, South Korea, India and Iran. Furthermore, these discrepancies in pharmacological effects of various aloe vera preparations may be due to several other factors, including a lack of consistency among studies in relation to standardization of aloe vera manufacturing process, dosage, duration of treatment, units of laboratory tests and races of the selected patients. Such heterogeneity confounds interpretation of statistical findings. We had initially planned to conduct subgroup analyses, however, there were not a sufficient number of trials to perform this analysis. Therefore, the random-effects model was adopted, although it cannot completely eliminate heterogeneity.

Despite these limitations, the present findings could provide useful information on the future research. First, insulin resistance is thought to be a key factor in the development of diabetes; unfortunately, insulin did not appear in a large number of studies outcomes. Second, lifestyle factors, such as food intake and physical exercise, are very important aspects of blood glucose control. However, the difference in the average daily caloric intake and level of physical activity undertaken by study participants was unclear. Most studies lacked objective outcome measures to estimate the extent to which these variations influenced the outcome of the study result. Therefore, focusing on these supplementary clues may be useful in future research studies on the topic. Third, not all of these included aloe vera preparations were assumed to be identical in the composition and biological activity they possessed, which might lead to the discrepancies in the glucose-lowering effect. The variation in daily dosages makes it difficult to determine the minimum effective dose of aloe vera that can cause a blood glucose reduction. Future studies should focus on the effects of aloe preparations, dosage, and the part of the plant used. Finally, the common adverse effects of aloe vera supplementation are abdominal pain, cramping, and muscle weakness [8,9]. However, only one study briefly described the adverse reactions of subjects in the aloe vera group [19]. The selected trials administered aloe vera for 6–12 weeks. Considering the fact that these studies were of short duration, the safety of long-term aloe vera intake seems uncertain. Thus, it is essential for investigators of future trials to incorporate surveillance time frames into the clinical trials to monitor any medium- and long-term adverse events associated with the use of aloe vera.

## 5. Conclusions 

In conclusion, the currently available data showed that aloe vera might reduce the levels of FBG, HbA1c, triglyceride, TC and LDL-C, and increase the levels of HDL-C in prediabetes and early non-treated diabetic patients; however, limited evidence exists about the safety of aloe vera, given the small RCTs, poor quality of RCTs included, and the considerable heterogeneity seen in the study results, the magnitude of this effect is small and the clinical relevance is uncertain. Large-scale, multi-center and placebo-controlled long-term trials should be rigorously designed to substantiate the current findings and to investigate the long-term effects of aloe vera supplementation on managing prediabetes and T2DM.

## Figures and Tables

**Table 1:** Characteristics of studies included in the meta-analysis.

Study	Main Diagnoses of Study Participants	Mean FBG (mg/dL)	Mean HbA1c (%)	Mean BMI (kg/m^2^)	Gender (Male/Female)	Randomized/Analyzed	Daily Dosage (Formulation)	Treatment Duration (Weeks)	Main Outcomes	Adverse Events	Control for Lifestyle Factors	Randomization Appropriate	Allocation Concealed	Sample Size Determined	Groups Similar at Baseline	Blinding	ITT Analysis
Yongchaiyudha, 1996 [17]	Early untreated diabetes	250.7	Unclear	Unclear	50/22	72/70	Two tablespoonful (juice)	6	Glucose and lipid profile	Unclear	Unclear	Unclear	Unclear	Unclear	Yes	Single blinding	Unclear
Devaraj, 2013 [18]	Prediabetes	109	5.9	34.7	15/29	45/44	1.0 g (capsules)	8	Glucose and lipid profile	Unclear	Normal diet and exercise at least 100 min per week	Unclear	Unclear	Unclear	Yes	Double blinding	Unclear
Choi, 2013 [19]	Prediabetes and early untreated diabetes	116.2	6.2	27.4	96/40	136/122	2.8 g (capsules)	8	Glucose, lipid profile and obesity-related biomarkers	One adverse event	Unclear	With the randomization code generated by software	Unclear	Unclear	Yes	Double blinding	Inadequate
Choudhary, 2014 [20]	Untreated diabetes	130.8	Unclear	Unclear	90/0	90/90	0.2 g (powder)	12	Glucose, lipid profile and blood pressure	Unclear	Unclear	Unclear	Unclear	Unclear	Yes	Unclear	Unclear
Alinejad-Mofrad, 2015 [21]	Prediabetes	111.1	6	28.2	21/49	72/70	1.0 g (capsules)	8	Glucose and lipid profile	Unclear	Normal diet and exercise	Blocking randomization	Unclear	Unclear	Yes	Double blinding	Unclear

BMI: body mass index; FBG: fasting blood glucose; HbA1c: hemoglobin A1c; ITT: intention to treat.

## Abbreviations

The following abbreviations are used in this manuscript:

CI	Confidence interval
FBG	Fasting blood glucose
HbA1c	Hemoglobin A1c
HDL-C	High density lipoprotein-cholesterol
ITT	Intention to treat
LDL-C	Low density lipoprotein-cholesterol
RCT	Randomized clinical trial
SMD	Standardized mean difference
T2DM	Type 2 diabetes mellitus
TC	Total cholesterol
WMD	Weighed mean difference
